# Surface Quality Optimization in Micromachining with Cutting Tool Having Regular Constructive Geometry

**DOI:** 10.3390/mi13030422

**Published:** 2022-03-08

**Authors:** Catalin Gabriel Dumitras, Dragos Florin Chitariu, Florin Chifan, Cristina Georgiana Lates, Mihaita Horodinca

**Affiliations:** Faculty of Machine Manufacturing and Industrial Management, Technical University Gheorghe Asachi of Iasi, 700050 Iasi, Romania; catalin-gabriel.dumitras@academic.tuiasi.ro (C.G.D.); florin.chifan@academic.tuiasi.ro (F.C.); cristina-georgiana.lates@student.tuiasi.ro (C.G.L.); mihaita.horodinca@academic.tuiasi.ro (M.H.)

**Keywords:** regular constructive geometry, micro-cutting, surface quality, optimization, fractional factorial design

## Abstract

In this paper we studied the influence of micromachining parameters on processed surface quality. Usually in discussions about micro-cutting or micromachining, the grinding or diamond turning processes are considered. Cutting tools used in the mentioned processes do not have regular constructive geometry and, in this case, it is difficult to use constructive geometric parameters such as clearance angle α or rake angle γ to optimize the quality of the machined surface. In order to determine the influence of the cutting tool’s constructive geometry on the hardness of the machined material, we used a fractional factorial design of a centered and rotatable type 2^6−1^. A mathematical model based on five independent cutting parameters was created that allowed optimization of surface quality based on obtained roughness. The results can be applied in micromilling or microturning.

## 1. Introduction

**Figure 1:**
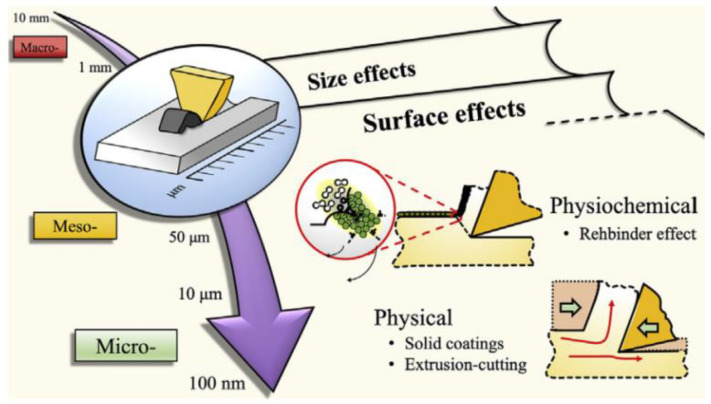
Indicative limits for different types of metal cutting [6].

The increasing complexity of new products and the increasing demand for obtaining high precision in conditions of productivity has imposed new technology and optimized cutting tools [1,2]. Rapid advances in technology have enabled the possibilities for superior accuracies in machine tools to unlock the potentials of ultraprecision machining with an accuracy tolerance band between 5 and 50 nm [3].While a wide variety of material removal processes exists like the abrasive jet process, in finishing processes, mechanical cutting (i.e., milling, turning, etc.) remains one of the favored processes in manufacturing lines, especially in the aerospace and automotive industries [4,5]. The graphic presented in Figure 1 establishes the limits that define three types of cutting: macro-, meso-, and micro-cutting.

Micro-cutting, as suggested by the terminology, involves uncut chip thicknesses, product dimensions, and tool geometry at the micrometric scale (≤100 μm), although the definition can vary up to 1000 μm [7].

**Figure 2:**
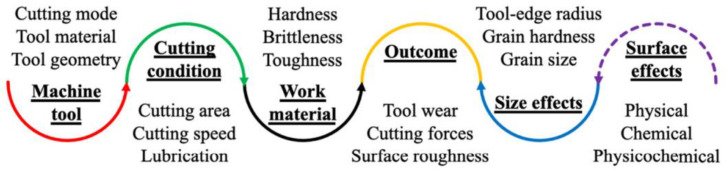
Overview of micro-cutting considerations [6].

Micro-cutting is mostly used in finishing and superfinishing processes which represent one of the most important steps in the manufacturing of a product because during this step, final conditions are obtained, imposed by the project and the utility of the product. The tolerance and quality of the manufactured surface becomes important. Micro-cutting considerations as shown in Figure 2 extend beyond the typical assessment of cutting conditions, machine tool vibrations, and tool wear [8]. At this level of machining, several micro-effects exhibit considerable influence on machining performance such as the transition in material deformation modes from ploughing to rubbing at a lower tool-edge sharpness to produce superior surface finishing on magnesium [9], aluminum, and copper alloys [10].

As seen in Figure 2, the cutting process is complex and there are a large number of parameters that can influence the results [11]. This can be an impediment to optimizing the quality of the obtained surface. Furthermore, if the process of micro-cutting is obtained by using abrasive particles, the process is even more difficult to optimize because geometry varies from one particle to another and it is difficult to establish a relationship between cutting parameters and the roughness of the machined surface [12,13].

**Figure 3:**
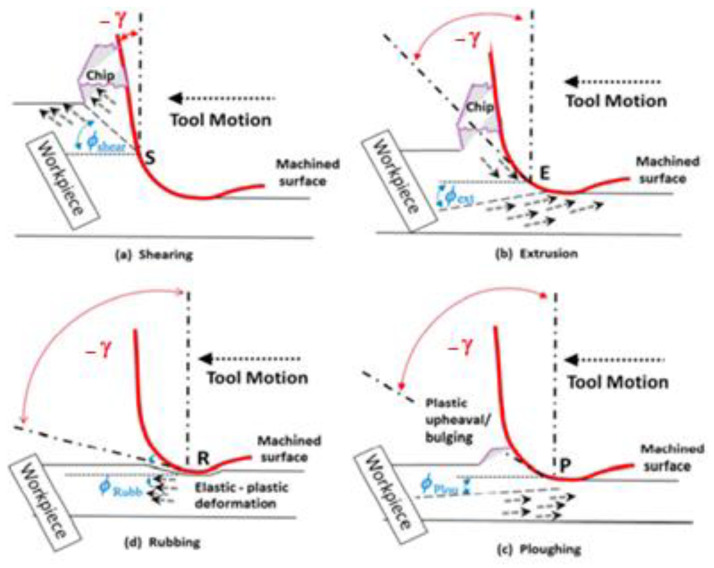
Material deformation mechanisms under effective varying rake angles: (**a**) shearing, (**b**) extrusion, (**c**) ploughing, and (**d**) rubbing [5].

The micro-cutting process is influenced also by the depth of cut values with respect to the cutting edge radius. With respect to the depth of cut values, we can have different cutting mechanisms as shown in Figure 3 [14,15,16]. If the value of cut depth is larger than the cutting edge radius value, a shearing mechanism appears in chip formation (Figure 3a). If the cut depth value is comparable to the cutting edge radius, there is a mechanism of chip formation based on extrusion (Figure 3b). If the cut depth value is much lower than the cutting edge radius, practically no cutting process occurs and it is replaced by a process of hardening of the top surface layer (Figure 3c). We can also observe that the rake angle varies with cut depth value while the cutting edge radius is constant, increasing in absolute value with decreasing depth of cut.

Alongside the cutting parameters, to achieve high quality machined surfaces, the impact of various correction factors in Computer Aided Manufacturing (CAM) programming must be also considered [17]. Various probabilistic techniques such as simulated annealing for approximating the global optimum of a given function can be used in the modeling and optimization of surface roughness [18].

The above studies have shown the complexity of a micro-cutting-process study. There are many machining parameters together with cutting tool geometry that can influence the quality of the machined surface. Therefore, we investigated the cutting performance of milling cutters with regular constructive geometry on surface roughness using experimental verification, and established a rotatable, centered, and fractional test in order to analyze the influence of different parameters such as clearance and inclination angles and the workpiece hardness on the roughness of the machined surface. Finally, we obtained the optimal parameter combination for the micro-textured ball-end milling cutter with a blunt round edge. This study aimed to provide the foundation for improving the efficiency of machining of alloyed steel using a regular constructive geometry cutter.

## 2. Materials and Methods

**Figure 4:**
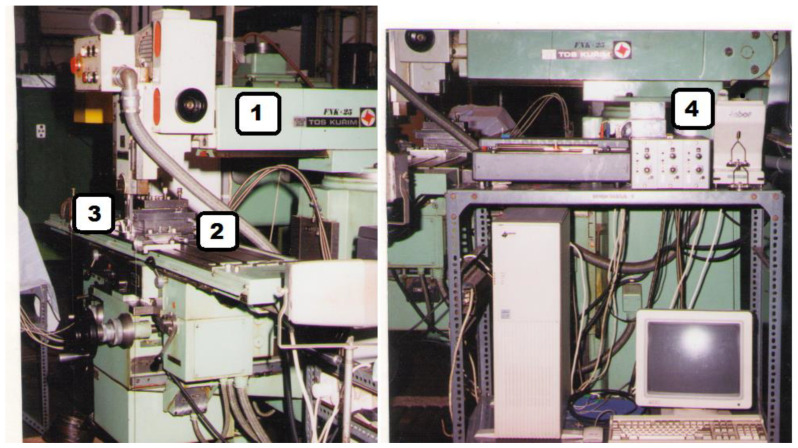
Experimental stand: (1) milling machine TOS KURIM FNK 25; (2) Multicomponent dynamometer KISTLER 9257 A; (3) Cutting tool; and (4) Multichannel charge amplifier Type 5080Axx3x001.

In order to determine the parameters of the micro-cutting process, we used an experimental stand that contained a clamping fixture for the workpiece mounted on a KISTLER 9257 A dynamometer, which can measure the cutting force in three directions. The whole assembly was mounted on the table of the TOS KURIM FNK 25 milling machine. This machine is equipped with a head that can be operated as a mortising machine with 5 speed steps: 50, 70, 100, 140, and 200 double stroke per minute or as a milling machine. The following measurement chain was used to acquire the data: KISTLER coaxial cables type 1610 B10 and KISTLER 5008 type amplifier, both for each measuring channel, analog-to-digital converter mounted on a PC computer, program data acquisition that could read process data from two channels with force on the direction of the M_z_ axis (main cutting force) and on the direction of the M_x_ axis (cutting force on the direction of the working feed). The experimental stand is presented in Figure 4 [19].

**Figure 5:**
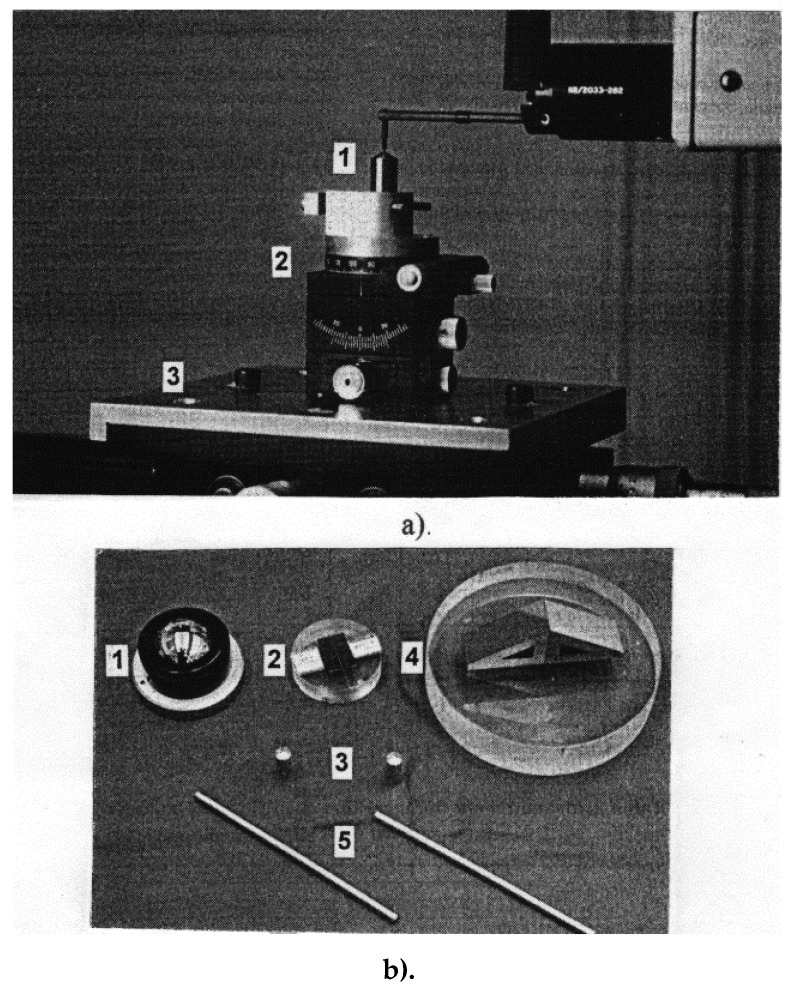
Cutting edge radius measuring device: (**a**) Talysurf 120L apparatus and (**b**) calibers—a steel ball with a diameter of 22 mm (1), a steel wire of 204.01 mm (2), two pieces of rubber of 198.71 mm (3), a piece of steel with a dihedral angle of 120 (4), and two steel bars (5).

In order to measure the cutting edge radius of the insert that was obtained by sharpening the insert, Talysurf 120L was used, as presented in Figure 5.

**Figure 6:**
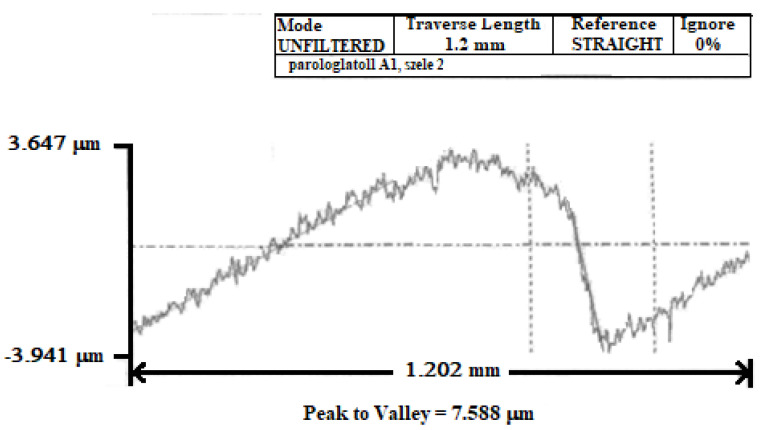
Measured radius on Talysurf 120 L.

Using this device, we measured the cutting edge radius of the insert. The results are presented in Figure 6.

Five tests were performed at different points on the cutting edge of the same cutting tool, and the measured values of the cutting edge radius were between 9 and 18 µm.

**Figure 7:**
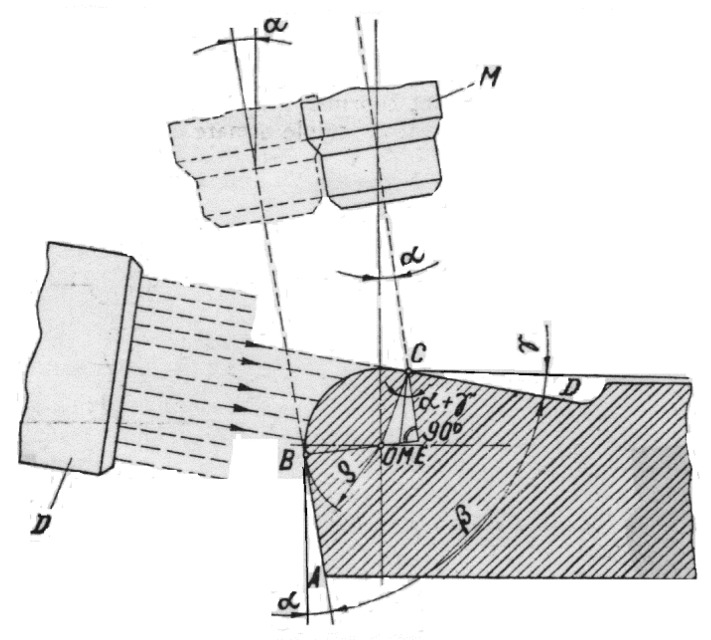
Double microscope Linnik–Schmaltz cutting edge radius measuring, where: α—clearance angle; γ—rake angle; ρ—cutting edge radius; β—sharpening angle; M—objective lens; and D—light source.

In order to confirm the measured values, we used the double microscope Linnik–Schmaltz as a second equipment and performed indirect measuring. In Figure 7, we present why this method was indirect. The microscope has a light source D that illuminates the insert at the radius level. The ocular of the microscope M is inclined with the value of the clearance angle α and it is moved from point B to C. The radius was calculated with the following equation:

(1) $$\rho =\frac{\mathrm{BC}}{1+\sin(\alpha +\gamma )}$$

The obtained values were between 9 and 12 µm.

**Figure 8:**
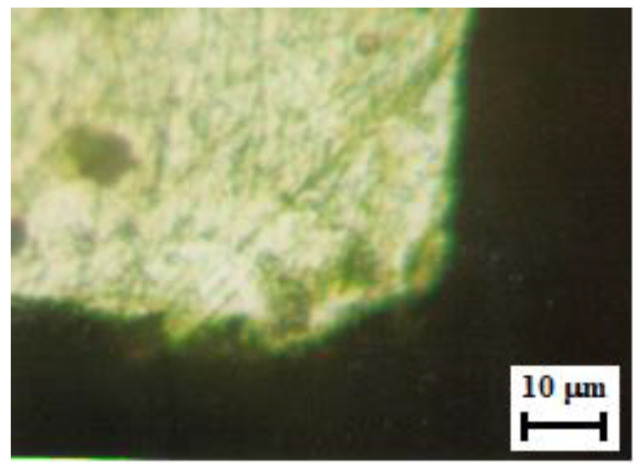
Cross-section image from the cutting insert.

**Figure 9:**
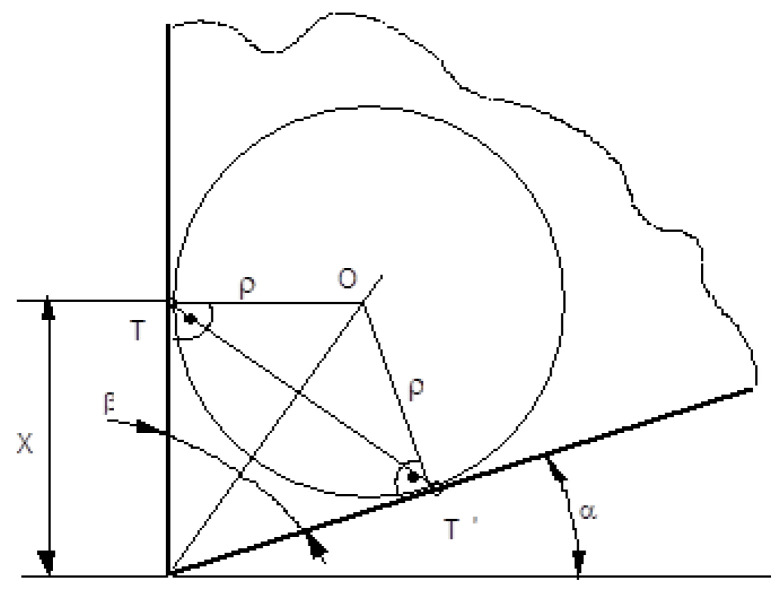
Geometric schematic for radius calculus.

The third method used to establish the values of the radius obtained by sharpening was a direct method. We made a cross-section through the insert and measured the radius on an image obtained from the microscope. The image was enhanced 400× (Figure 8). The equation for obtaining the cutting edge radius was established using the geometric construction presented in Figure 9.

The equation for cutting edge radius determination was:

(2) $$\rho =X\cdot \mathrm{tg}\left(\frac{\beta }{2}\right)$$

The measured value was 12 µm.

The cutting edge radius was measured in order to define the influence of this radius on surface quality and the processed surface layer.

In order to determine the influence of the cutting process parameters on surface quality, we chose to use a fractional factorial design for the experiment that is centered and rotatable, namely type 2^6−1.^ This experimental design implied 32 experiments.

The objective function that was optimized was surface roughness R_a_. The optimization factors were:

- v—cutting speed, m/min;
- F_p_—radial force, N;
- α—clearance angle, rad;
- λ—inclination angle, rad; and
- δ_HB_—workpiece hardness, N/mm^2^.

Five values with a fix step Δp (−2, −1, 0, 1, and 2) were proposed for each variable parameter.

Considering the optimization factor, the equation for the objective function became:

(3) $$R_{a}=C_{R_{a}}e^{a_{1}\mathrm{lgV}}e^{a_{2}F_{P}}e^{a_{3}\alpha }e^{a_{4}\lambda_{}}e^{a_{5}\delta_{\mathrm{HB}}}$$

Assuming the following notations: Y_1_ = lnR_a_; x_1_ = F_P_; x_2_ = log v; x_3_ = α; x_4_ = λs; x_5_ = δ_H_; and b_0_ = ln C_Ra_ in Equation (3), we obtained:

(4) $$Y_{1}=b_{0}+\sum_{i=1}^{i=5}b_{i}x_{i}$$

In (4), the coefficients of b_i_ are regarded as constants, which limits from the beginning the degree of approximation of the Y_i_ functions.

To increase the accuracy of objective function approximations, the literature [3] recommends developing functions in Taylor series around a convenient point (center of the experiment) and truncating them to a convenient number of terms, so that the approximation relationship becomes:

(5) $$Y_{1}^{*}=b_{0}^{*}+\sum_{i=1}^{i=5}b_{i}^{*}x_{i}+\sum_{i=1;i<j}^{i=4;j=5}b_{ij}x_{i}x_{j}+\sum_{i=1}^{i=5}b_{ii}x_{i}^{2}$$

In order to determine the coefficients in (5), the technique of designing experiments was used [20] which has the following advantages over other techniques of experimentation:

- Minimum number of experiences;
- High precision of the obtained mathematical model; and
- Equal and uniform distribution of experiences throughout the experimental field, so that the objective function obtained is defined throughout the studied field.

Given that the range of variation of the independent variables in (5) cannot be described by means of linear approximation (interaction effects cannot be highlighted), a composite (order of 2) experimental plan was used.

The necessary steps to implement such a program were as follows:

- Sorting variables that can significantly influence the results of the experiment (it is preferable to retain as many variables as possible because the insignificant variables are eliminated by subsequent statistical evaluation, and experiences in which those variables have changed are used to increase the accuracy of the answer);
- Selection of technologically allowed variation intervals for each variable;
- Choosing intervals of discrete points (called levels or experimental points) for evaluation of the variables, which form the experimental program (also called programming matrix);
- Determining the size of experimental error;
- Performing specified experiments from the program in a random order;
- Measuring the response of each experience;
- Statistical analysis of the collected data; and
- Interpretation of the technological meaning of the results of statistical analysis.

This type of program is obtained from a complete factorial program in order to estimate all coefficients of first-order terms and second-order interactions. This program is then supplemented with points that allow the estimation of square terms.

Not knowing the orientation of the response surface to the coordinates, a composite, centered, and rotatable experimental program was chosen with the property of equal standard deviation for all points at equal distances from the center of the experimental region.

For each independent variable, we considered five values where −1 and 1 were the limits of the considered interval, 0 was the center of the interval (this value must be considered because there are 6 experiments using these values using a centered factorial program), and −2 and 2 were outside the interval in order to verify if the determined interaction of the independent variables remain the same outside the considered intervals.

Variation intervals of the independent variables which characterize parameters of the working regime were established in accordance with the adopted experimentation strategy. In this sense, quantities have been provided by specialized literature for the extreme values of speed v, inclination angles λ, clearance angle α, and radial force F_p_, which place them at the limits (or even outside) of the recommended optimal range.

The values of the independent variables are presented in Table 1.

When establishing the hardness variation interval δ_HB_, the aim was to impose harsher working conditions than usual by using an alloy steel of type 40 Cr 10 [19], printed by applying heat treatments corresponding to five hardnesses. The experimental samples are presented in the form of bars with a rectangular section 12 mm × 10 mm, with a length of 200 mm.

The solutions for the system resulting from this experimental program (which represent the coefficients of the equation for estimating the response area for each objective function in coded coordinates) are given by (6) [20].

(6) $$b_{0}=0.159091\sum_{n=1}^{n=32}Y^{*}{}_{n}-\sum_{n=1}^{n=32}\left(\left(\sum_{i=1}^{i=5}x_{ic}^{2}\right)Y^{*}{}_{n}\right)\;b_{i}=0.041667\sum_{i=1;n=1}^{i=5;n=32}x_{ic}Y^{*}{}_{n}\;b_{ij}=0.0625\sum_{\begin{array}{l} i=1;n=1\\ i<j \end{array}}^{i=5;n=32}x_{ic}x_{jc}Y^{*}{}_{n}\;b_{\mathrm{ii}}=0.03125\sum_{i=1;n=1}^{i=5;n=32}x_{\mathrm{ic}}^{2}Y^{*}{}_{n}+0.002841\sum_{n=1}^{n=32}\left(\left(\sum_{i=1}^{i=5}x_{\mathrm{ic}}^{2}\right)Y^{*}{}_{n}\right)-0.034091\sum_{n=1}^{n=32}Y^{*}{}_{n}$$

## 3. Results

In the following section, we present the topography of the processed surface, measured in several directions.

**Figure 10:**
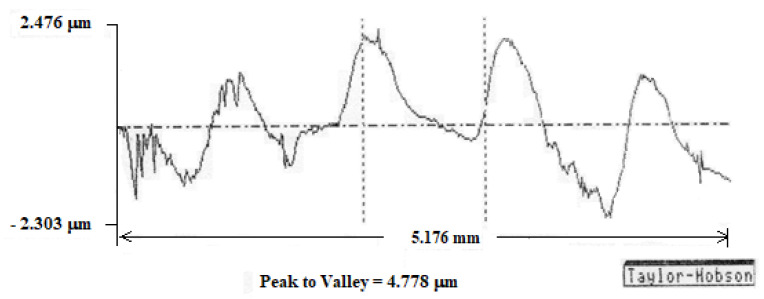
The topography of the manufactured surface, measured on Talysurf 120 L.

Measurements were made on a perpendicular direction to the machining marks. The results are shown in Figure 10, noting that the scales on the coordinate axes are different. We observed a periodicity of the profile which can be explained by the fact that the cutting inserts enter in the workpiece periodically and also by the dynamics of the cutting process.

**Figure 11:**
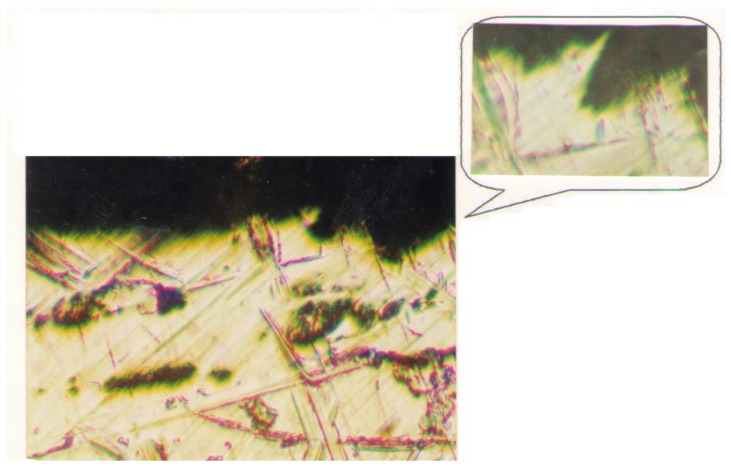
View manufactured surface with a chip root (×100); (a portion of the ×500 scale view is shown at the top).

In Figure 11, we present a cross-section made in a longitudinal section of the sample with hardness HB = 300. The root of a chip and the deep entrance of the insert in the workpiece can be observed. Again, this can be explained by considering the dynamics of the cutting process.

**Figure 12:**
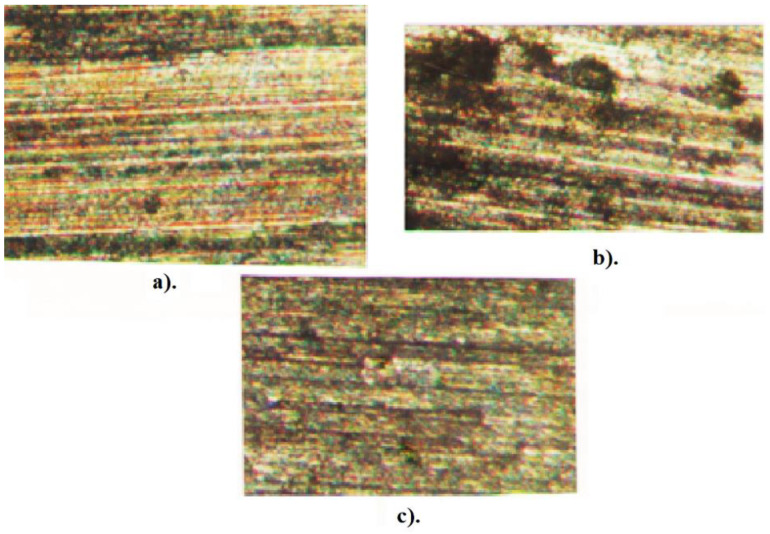
The topography of the manufactured surface: (**a**) workpiece hardness HB = 300; (**b**) workpiece hardness HB = 400; and (**c**) workpiece hardness HB = 200.

Figure 12 shows the appearance of the surface resulting from machining with a cutting tool having regular constructive geometry. It is clear that the first case of processing involves pure cutting while the second case involves multiple scratching (scraping) of the surface to be processed which has the effect of removing material. The vertical lines in Figure 12b represent traces of the cutting tool insert. Again in Figure 12, the chip root left by the cutting tooth can be observed by its dark color. Figure 12c shows the surface resulting from a manufacturing material with hardness HB = 400. In this case, the cutting process was unstable, with strong vibrations being introduced into the system. These vibrations led to a severely affected surface, with dark spots highlighting areas of impact between the teeth of the cutting tool and the blank.

**Figure 13:**
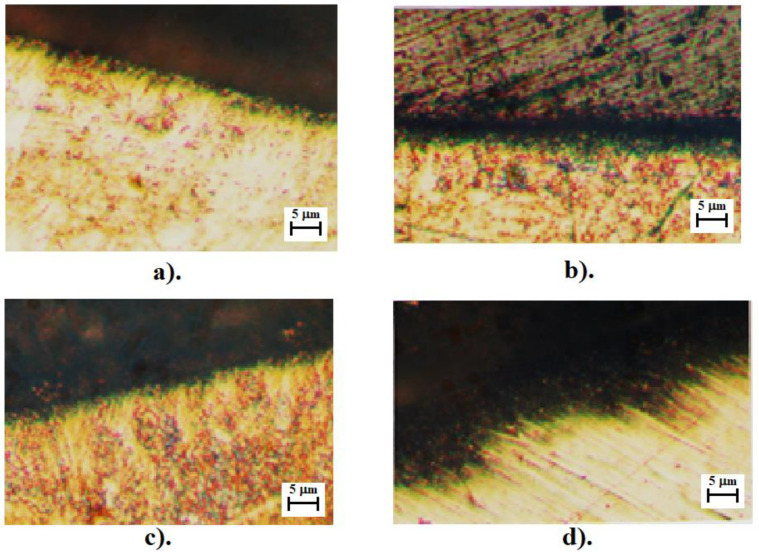
Depth of the surface layer: (**a**) machined workpiece—hardness HB = 200; (**b**) machined workpiece—hardness HB = 300; (**c**) machined workpiece—hardness HB = 350; and (**d**) machined workpiece—hardness HB = 400.

Additionally as mentioned, a study was performed on the surface layer that focused on measuring the depth of hardening. In this case, a comparative study was performed which led to the conclusion that in the case of processing with the considered type of tool, the depth of the affected layer is reduced by 30–80% (Figure 13). Figure 13c shows the surface layer for the sample with hardness HB = 400, with its study demonstrating through strong non-uniformity a strong level of vibrations and that the cutting tool did not work in optimal conditions.

In the following section, we present the mathematical model obtained by using the design of fractional factorial design of experiments 2^6−1^.

To increase the accuracy of the deduced mathematical model, all the coefficients were taken into account when establishing the regression functions, regardless of their significance level.

Consequently, the mathematical models of the objective functions resulting from the regression have the following form in coded coordinates:

Y_1_* = 0.370932 + 0.0577727 x_1_ − 0.0405179 x_2_ + 0.0864213 x_3_ − 0.052443 x_4_ − 0.0310602 x_5_ + 0.169519 x_1_ x_2_ − 0.0962358 x_1_ x_3_ − 0.166504 x_1_ x_4_ + 0.0728489 x_1_ x_5_ + 0.0930828 x_2_ x_3_ + 0.0921178 x_2_ x_4_ − 0.129986 x_2_ x_5_ − 0.148616 x_3_ x_4_ − 0.0997985 x_3_ x_5_ − 0.204017 x_4_ x_5_ + 0.0290287 x^2^_1_ − 0.0982925 x^2^_2_ − 00238559 x^2^_3_ − 0.163527 x^2^_4_ − 0.158981 x^2^_5_ (7)

In order to determine the regression equations of the objective functions in natural coordinates, a coordinate transformation was performed based on (6) and Table 1, according to the following relationships:

(8) $$\begin{array}{cc} \; & x_{1}=0.25\;F_{P}-4\\ \; & x_{2}=6.8433154\log v-2.0600364\\ \; & x_{3}=14.32394489\;\alpha -5\\ \; & x_{4}=11.45915493\;\lambda -2.99999977\\ \; & x_{5}=0.02\;\delta_{\mathrm{HB}}-6 \end{array}$$

Replacing (8) in (7), the mathematical models of the objective functions in natural coordinates take the form:

ln R_a_ = −16.8241 + 0.00498102 F_P_ − 1.88512 log v + 19.3102 α + 38.6419 λ + 0.059282 δ_HB_ − 0.344619 F_P_ α + 0.290018 F_P_ log v − 0.476997 F_P_λ + 0.000364245 F_P_δ_HB_ + 9.12428 αlogv + 7.22375 λ log v − 0.0177908 δ_HB_ log v − 24.3939 αλ − 0.0285902 α δ_HB_ − 0.0467572 δ_HB_ λ + 0.00181429 F_P_^2^ − 4.60313 log v^2^ − 0.489465 α^2^ − 21.4731 λ^2^ − 0.0000635924 δ_HB_^2^ (9)

In Equation (9), the independent variables are considered in the units of measurement presented in Table 1.

In order to determine the final form of the objective functions, of the constants and coefficients of (3), Equation (9) was used in which the terms were conveniently grouped. After performing the necessary calculations, the respective coefficients, and constants took the form shown in Table 2.

In order to validate the mathematical model, several tests were performed. Gruber test for identification of aberrant data was performed. The results for the proposed mathematical model in Equations (7) and (9) have values v_1_ = 1.27808 and v_2_ = 1.54715 that are inferior to critical values of v = 3.21, resulting in a 95% confidence level.

A second Shapiro–Wilk (S–W) test was performed in order to verify the normality of the experimental data string. The calculated value W = 1.39187 was compared to the tabular value of W = 0.788 for the S–W test, indicating with a 95% confidence level the acceptance of the normality of the data string hypothesis.

A Fischer (F) test was performed in order to verify the adequacy of the mathematical model. The calculated value F of the test must be less than the critical value FT indicated by the literature. The calculated value was 3.39199 which is lower than the value 4.95; therefore, the condition was fulfilled for an alpha coefficient of 0.05, i.e., the confidence level of the results was 95%.

## 4. Discussion

**Figure 14:**
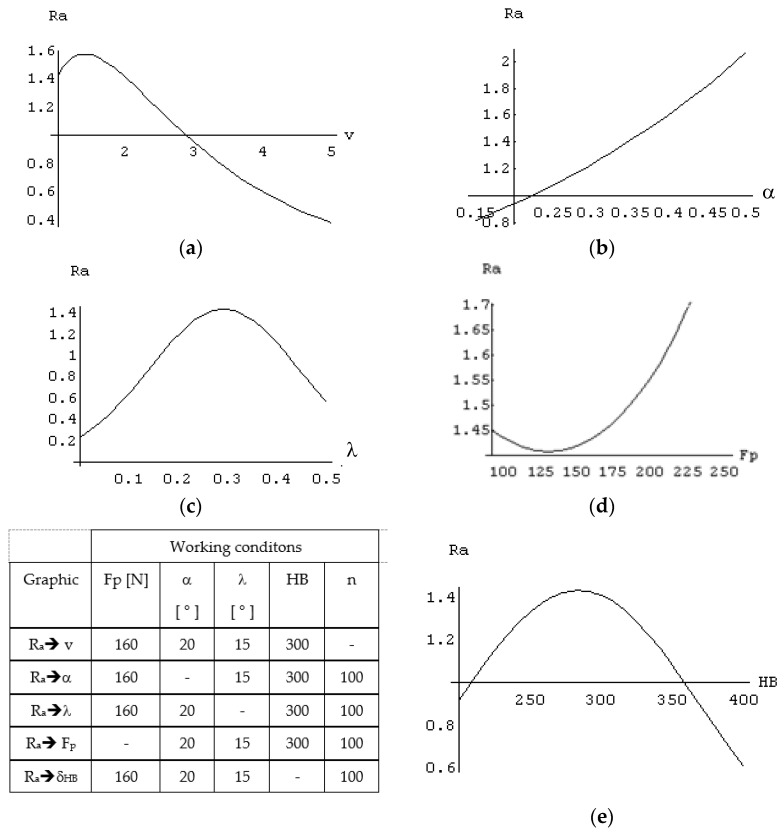
Influence of the optimization factors on objective function “roughness R_a_”: (**a**) speed influence on roughness (μm); (**b**) afla influence on roughness (μm); (**c**) lambda influence on roughness (μm); (**d**) Fp influence on roughness (μm); and (**e**) hardness influence on roughness (μm).

In the following section, we present dependencies of the objective function in relation to each optimization factor (Figure 14).

From graphs presented in Figure 14, one can conclude that the optimization factor “cutting speed” complex influences the roughness of the processed surface, and we observe a slight decrease in this parameter with increase in the cutting speed. However, at low speeds, there is a tendency to form adhesions to the edge which leads to the increase in roughness in the first phase, given that roughness decreases with the increase in cutting speed. In the case of the optimization factor “clearance angle α”, a pronounced increase in roughness was observed with the increase of this angle. This can be explained by the fact that there is a close connection between the clearance angle α and the back rake angle γ, so the increase of the clearance angle leads to high negative values for the back rake angle. This leads to high stresses required for cutting (complex cutting) and vibrations in the system. The optimization factor “inclination angle λ” also has an influence with a higher degree of complexity. In the first phase, a decrease in roughness is observed with an increase of this angle, but once it reaches a certain value, there is improvement. The first part of the graph is explained by an increase in the cutting effort, although a proper evacuation of the chips is ensured. Once the angles reaches a certain value, there is a decrease in roughness, and all these factors are determined by an increase of the contact length between the total active edge of the cutting tool and the blank along with an increase of the angle of inclination of the blade. For the same radial force F_p_, at a distribution of this force on a longer contact length, there is a decrease in specific pressure; from a certain angle, an exit process for the cutting teeth and a strong friction phenomenon between the cutting tool and the resulting roughness takes place. The optimization factor “radial force F_p_” also is complex in the way of variation of the roughness. Thus, with the increase in radial force, there is an insignificant decrease, although from a certain value it acquires a strong ascending character. This can be explained by the fact that this radial force directly determines the depth of cut, which in turn causes an increase in roughness as radial force increases. In the first phase, the optimization factor “HB hardness” leads to an increase in roughness motivated by the increase in cutting efforts and implicitly in vibrations in the system. Over a certain threshold a decrease in roughness is observed and, with the increase in hardness, the specific radial force necessary for processing can no longer be supplied by radial force F_P_; this results in a friction phenomenon instead of a cutting type, and leads to an improvement in roughness but has a negative effect on the whole processing.

**Figure 15:**
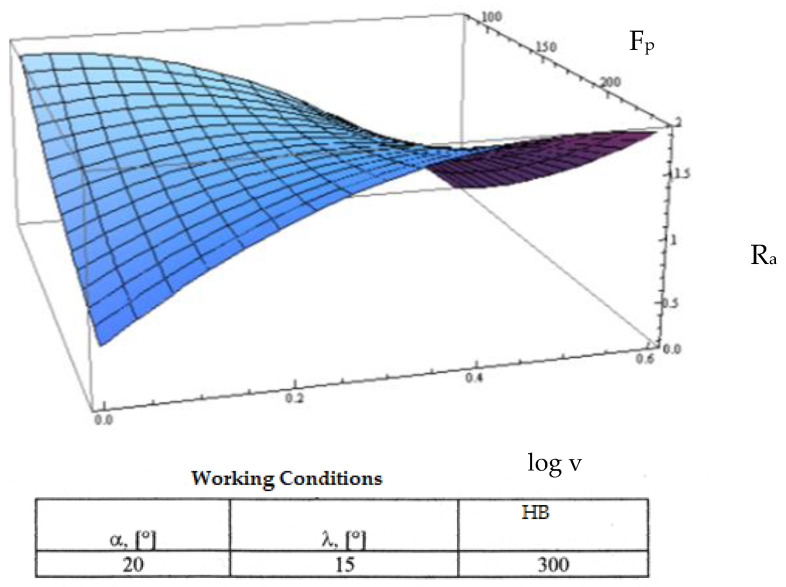
Influence of the cutting speed v and radial force F_p_ on roughness R_a_.

**Figure 16:**
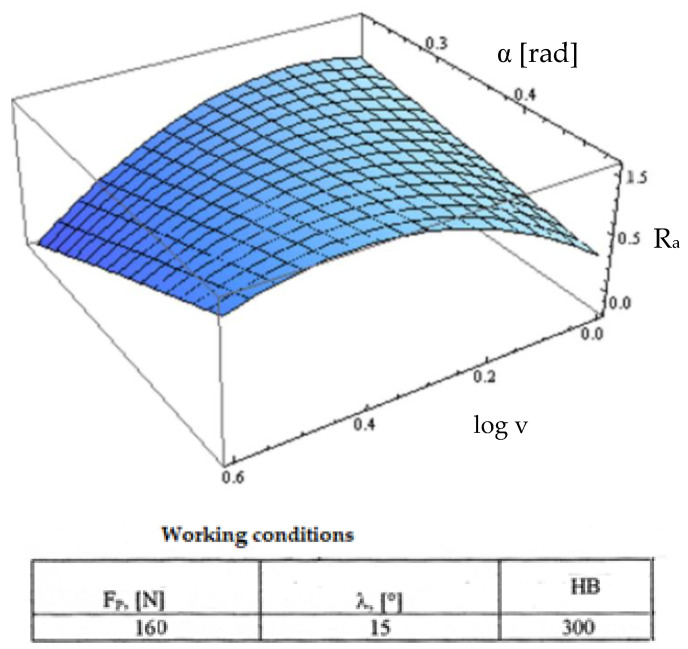
Influence of the clearance angle and cutting speed v on roughness R_a_.

**Figure 17:**
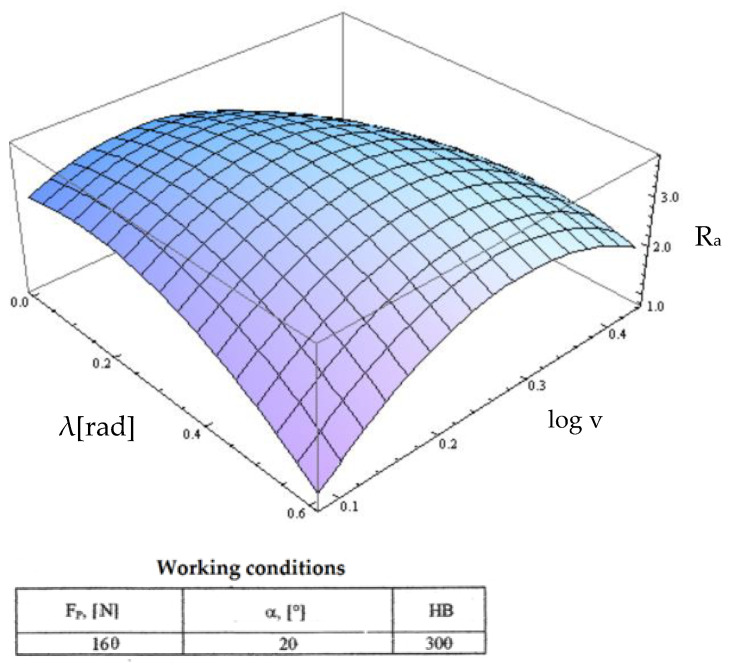
Influence of the inclination angle and cutting speed v on roughness R_a_.

**Figure 18:**
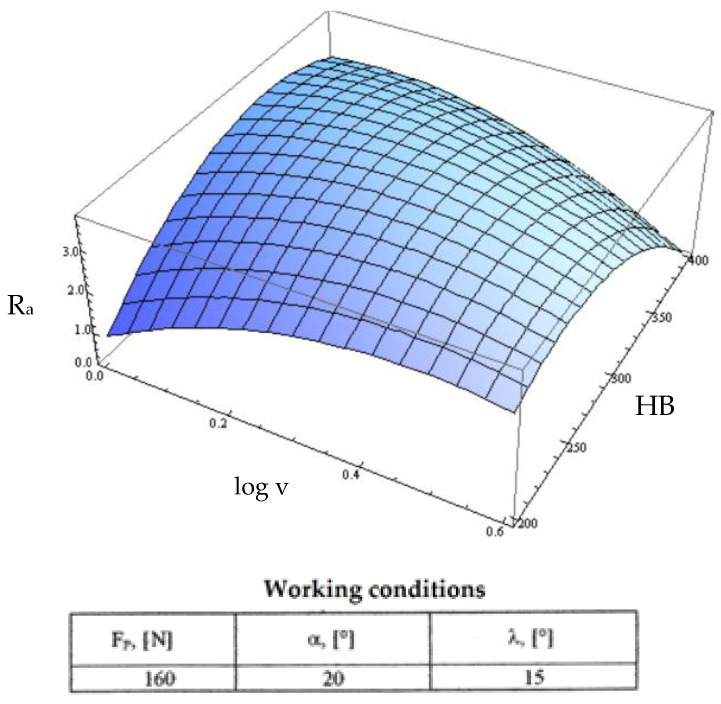
Influence of the hardness HB and cutting speed v on roughness R_a_.

**Figure 19:**
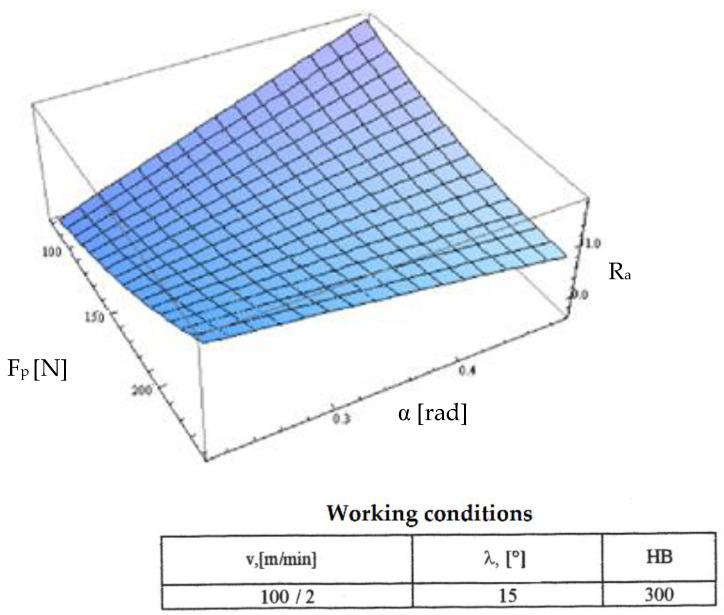
Influence of the clearance angle and radial force on roughness R_a_.

**Figure 20:**
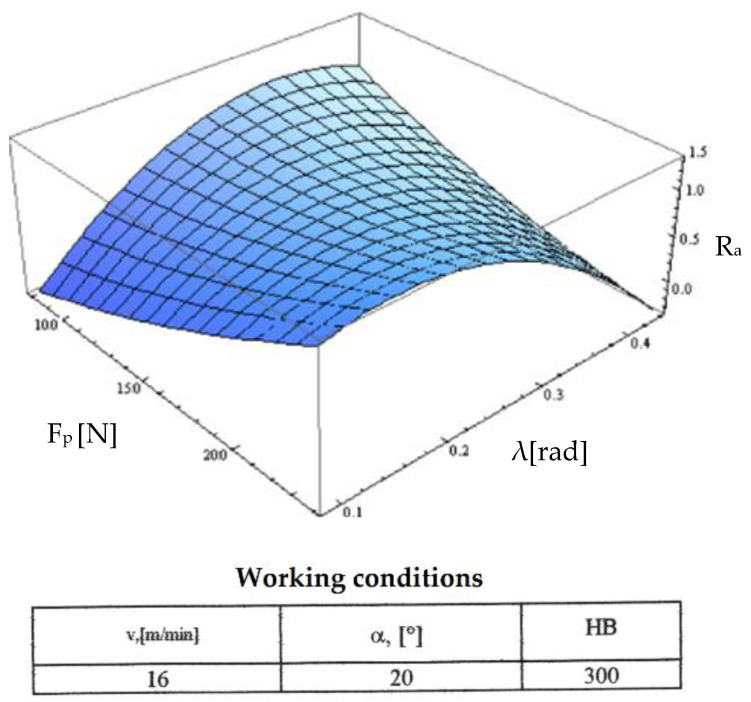
Influence of the inclination angle and radial force on roughness R_a_.

**Figure 21:**
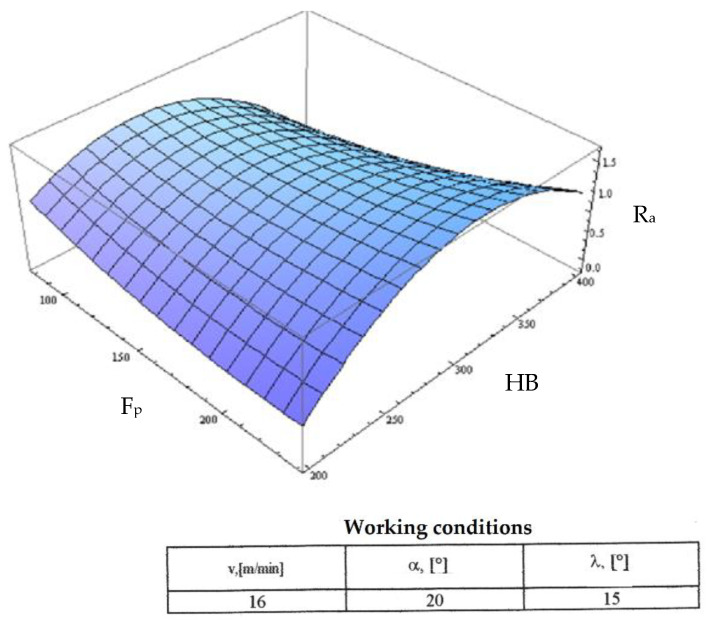
Influence of the hardness and contact force on roughness R_a_.

**Figure 22:**
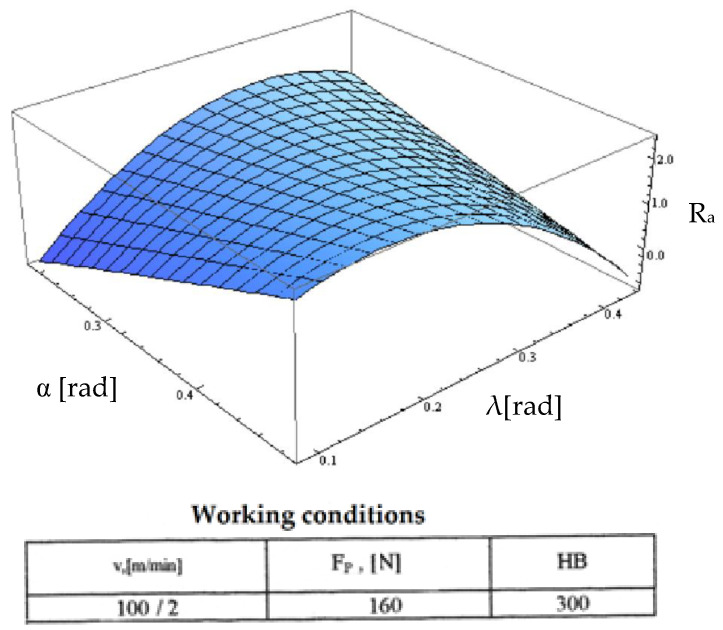
Influence of the clearance angle and inclination angle on roughness R_a_.

**Figure 23:**
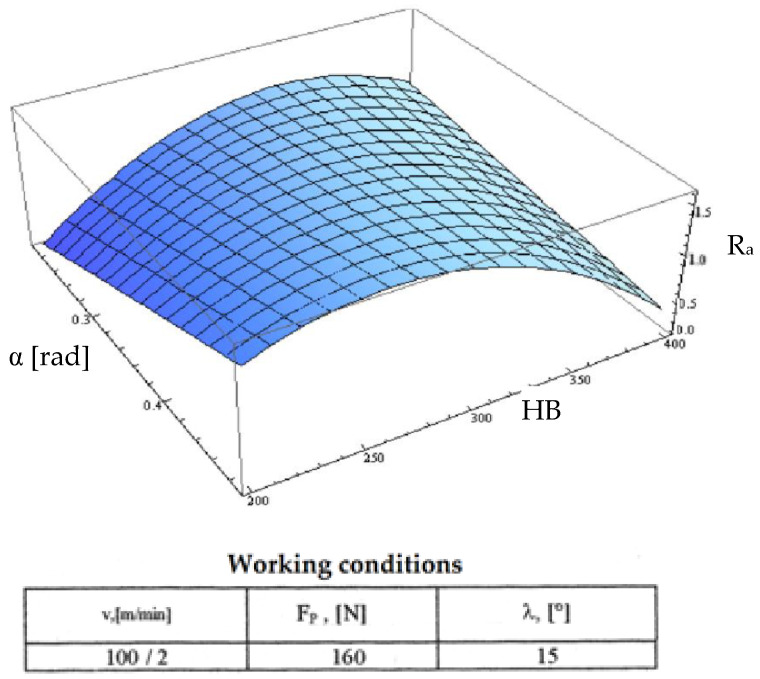
Influence of the clearance angle and hardness on roughness R_a_.

**Figure 24:**
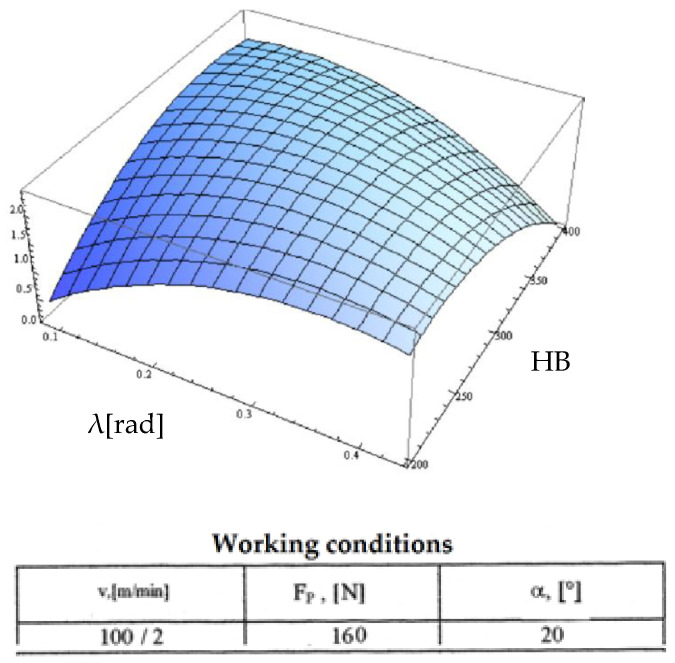
Influence of the inclination angle and hardness on roughness R_a_.

Starting from the determination and explanation of some influences between the optimization factors and the objective function “roughness”, these dependencies were processed in a two-dimensional space. We move onto the complex influence of the optimization factors in the three-dimensional space. As previously indicated, three optimization factors were kept constant, assigning the values from the center of the experiment field, and the other two factors were varied in the intervals established in the programming matrix of the factorial experiment. By the established method, we obtained a series of 10 graphs presented in Figure 15, Figure 16, Figure 17, Figure 18, Figure 19, Figure 20, Figure 21, Figure 22, Figure 23 and Figure 24.

These graphs are designed to have both a spatial view of the results and their two-dimensional presentation by means of isohypses or constant level curves, made by the intersection of graphs with planes parallel to the basic plane. This way of representation was possible by using the programming packages in mathematical language Mathematica ver.8, and the program necessary for this representation is attached to the doctoral thesis in the appendix.

Figure 15 shows the variation of the objective roughness function in relation to the cutting speed and the radial force F_p_. From this graph, it can be seen that the radial force has a greater influence on changing the way of variation of the roughness depending on the cutting speed and for high values of the radial force. The explanation of this phenomenon is that higher values of the cutting speed and the radial force induce a substantial increase in the cutting force with the corresponding consequences.

In Figure 16, the value of the roughness function in relation to the cutting speed is decreased for the whole surface, with the specification that roughness worsens for high values of the clearance angle α and low values of the cutting speed. At these values of the laying angle, we observed pronounced negative values of the clearance angle α, indicating high efforts and the possibility of vibrations with negative consequences on the quality of the processed surface.

Figure 17 shows the variation of the roughness function as a function of the variation of the cutting speed and the inclination angle λ. In this case, the cutting speed practically only amplified the influence of the angle of inclination at its low values and attenuated this influence for high values. The angle of inclination retained its influence as noted in Figure 15.

It should be noted in Figure 18 the same type of conjugate influence in which the cutting speed amplifies the phenomena to its small values. However, at high hardness of the blank, we observed that the cutting speed constantly decreased without the slightly increasing threshold shown in Figure 14. The increase in roughness continuing up to a certain hardness value can be explained by the increase in cutting efforts followed by an amplification of the vibratory phenomena. After a certain hardness value, there was a slight improvement of the roughness, explained by the fact that the specific force manifested in the cutting area increases while the friction phenomena amplified and thus led to better roughness.

A conclusion that can be deduced from the presented graphs is that the amplification of variation of roughness dependent on the optimizing factors (for all optimization factors) manifested in low cutting speeds.

Figure 19 presents the influence of optimization factors in which the main role is held by the radial force F_p_. This graph shows the combined influence of the optimization factors “contact force F_p_” and “clearance angle α”. We observed that at high values of the radial force and low values of the clearance angle the roughness worsened, explained by the fact that the cutting force increases as cutting depth increases; therefore, the efforts in the system increase along implicitly with vibrations and inconstant cutting. There is a possibility that the teeth of the cutting tool can penetrate the semi-finished product at one part and exit the cutting in another area). At high values of the laying angle and low values of the cutting force, we observed the worsening of the objective roughness function as a result of a dependency between the clearance angle α and the back rake angle γ that led to pronounced negative values for the clearance angle at high values of the seating angle. A decrease of the values in the objective roughness function results from the negative effects of negative geometry (complex cutting) which are counteracted by a higher specific radial force that maintains constancy of the cutting process with positive consequences.

Figure 20 presents the combined influence exerted by the optimization factors “F_p_ contact force” and “inclination angle λ”. In this graph, we can see the character of the variations presented in Figure 14 for both small values of the radial force and the angle of inclination of the edge. For high values of both optimization factors, there was a pronounced decrease of the values in the objective roughness function explained by the fact that the inclination angle increases as the contact length between the active and semi-finished edge increases and it also increases the specific force with positive effects on the cutting process in terms of its constant character.

Figure 21 presents the combined influence of the optimization factors “radial force” and “HB hardness” on the material to be processed. We observed that for low hardness of the material, the increase in radial force led to a slight decrease of the values of the objective function as a result of the vibratory phenomena in the system. As the hardness of the material increased, the repelling phenomenon of the material intensified, which led to a worsening of the roughness. At high values of material hardness (400 HB), we observed practically no machining for small radial forces but only friction and amplified vibrational phenomena in the system with increasing radial force, demonstrated in practice by increased acoustic phenomena and an intense dynamism of the measured cutting force.

Figure 22 shows the influence of the optimization factors “clearance angle α” and “inclination angle λ”. In this case, both optimization factors retained their influence on the roughness objective function, their entire range of variation, and on that presented in Figure 14. However, we observed that for small values of the seating angle, the maximum value of the influence of the inclination angle λ changed to higher values. This can be explained by the fact that at low values of the clearance angle, we obtained small negative values of the back rake angle, making the cutting efforts smaller and the specific radial force required for cutting lower. Then, with the increase of the contact length between the cutting tool and the semi-finished product that was modified by increasing the angle of inclination of the cutting edge, a maximum of this specific force could be reached for a higher value of the contact length, implying a greater angle of inclination of the edge.

Figure 23 shows the combined influence of the optimization factors “clearance angle α” and “hardness HB”. The influence of the seating angle was dictated both by the frictional forces in the system and by the back rake angle γ with which it is directly interdependent. With the increase of the setting angle and increase in hardness, the objective roughness function acquired high values given that roughness decreases from a certain value of hardness, in which case the cutting tool does not work, producing only a friction phenomenon.

Figure 24 shows the combined influence of the optimization factors “inclination angle λ” and “HB hardness”. The evolution of the variations of the objective roughness function is approximately the same as the one from the previous point. However, the explanation is modified, this time discussing the change in the active length between the cutting edge and the semi-finished product. It should be noted that as the hardness of the blank increases, the specific radial force required for cutting increases. In summary, the cutting process is inconsistent for the increases manifested on the two optimization factors. However, the F_p_ contact force is no longer enough from a certain hardness value with the cutting teeth gradually coming out of the cutting, which leads to the phenomenon of friction between the cutting tool and semi-finished product with a positive effect on the roughness value but a negative effect on other parameters.

Based on the above discussion, we determined an optimal solution which is represented by the intervals of variation of the optimization factors that can ensure the satisfaction of a high level of the main objective function and, at the same time, obtain possible minimum values of the secondary functions (Table 3).

## 5. Conclusions

The analysis of the two-dimensional sections of the response surfaces highlighted the following:

- The response surfaces of the functions “roughness Ra” have complex forms due to the important and sometimes contradictory influences of the independent variables on the objective functions;
- The response surfaces of the “roughness Ra” function have the shape of “elliptical hills”, at which the minimum points are not always within the considered range. Moreover, it is difficult to detect the minimum area of the cutting forces for which the cutting tool works properly, given that the cutting process easily becomes unstable when micro-cutting due to the high specific forces manifested in the working area, which can lead to the exit of the cutting tool from the cutting; and
- The contradictory influence of the independent variables makes it impossible to locate their domain that would satisfy to a maximum or minimum all the objective functions.

The adopted multicriteria optimization method was adapted to the shapes of the determined response areas and was based on the following strategy:

- Considering that the shape of the response surfaces of the main objective function (roughness or productivity) allows the identification of intervals of variation of the optimization factors in which it remains constant, it was determined at the beginning the experimental field which ensured the satisfaction of this criterion at a certain level;
- The limits of this field (within which the optimal global solution is located) were used to condition the values of the other objective functions; the conditioning was done both from the perspective of the intervals of definition for the independent variables and the extreme values that these functions can take.

## Figures and Tables

**Table 1:** The intervals of the independent variables.

Variables	−2	−1	0	1	2	Δp
x_1_ = F_p_ [N]	8	12	16	20	24	+4
n (c.d./min)	50	70	100	140	200	-
v (m/min)	1	1.4	2	2.8	4	-
x_2_ = log v	0	0.1461	0.3010	0.4471	0.602	0.1461
Clearance angle α [°]	12	16	20	24	28	-
x_3_ = α [rad]	0.20943	0.27925	0.34906	0.4188	0.48869	0.0698
Inclination angle λ [°]	5	10	15	20	25	-
x_4_ = λ [rad]	0.08726	0.17453	0.26179	0.34906	0.43633	0.08726
x_5_ = δ_HB_	200	250	300	350	400	+50

**Table 2:** Expression of the objective function coefficients.

Objective Function	Exponent	Expression
	a_1_	−1.83 + 9.13 α + 0.29 F_p_ − 0.02 δ_HB_ + 7.23 λ − 4.6 log v
R_a_	a_2_	0.01 − 0.35 α + 0.01 F_p_ + 0.0004 δ_HB_ − 0.48 λ
C_Ra_ = e^−16.83^	a_3_	19.31 − 0.49 α − 24.34 λ
	a_4_	38.64 − 21.47 λ
	a_5_	0.06 − 0.03 α − 0.0007 HB − 0.047 λ

**Table 3:** Optimized intervals of parameters.

Objective Function	Parameters	F_p_ [N]	Clearance Angle α [rad]	Inclination Angle λ [rad]	Hardness HB
Roughness R_a_	R_a_→F_p_, V	[120 ÷ 250]	-	-	-
	R_a_→V, α	-	[0.15 ÷ 0.4]	-	-
	R_a_→V, λ	-	[0.1 ÷ 0.45]	-	-
	R_a_→V, HB	-	-	-	[225 ÷ 370]
	R_a_→F_p_, α	[110 ÷ 140]	[0.15 ÷ 0.45]	-	-
	R_a_→F_p_, λ	[120 ÷ 240]	-	[0.2 ÷ 0.4]	
	R_a_→F_p_, HB	[100 ÷ 225]	-	-	[220 ÷ 350]
	R_a_→α, HB	-	[0.35 ÷ 0.5]	-	[200 ÷ 350]
	R_a_→λ, HB	-	-	[0.2 ÷ 0.5]	[200 ÷ 350]
Optimized domain of working conditions		[120 ÷ 140]	[0.35 ÷ 0.4]	[0.2 ÷ 0.4]	[225 ÷ 350]

## References

[1] Bottin M., Cocuzza S., Massaro M. (2021). Variable Stiffness Mechanism for the Reduction of Cutting Forces in Robotic Deburring. Appl. Sci..

[2] Brinksmeier E., Gläbe R. (2001). Advances in precision machining of steel. CIRP Ann..

[3] Dornfield D., Lee D.E., Dornfeld D., Lee D.-E. (2008). Introduction to precision manufacturing. Precision Manufacturing.

[4] Egashira K., Furukawa T., Yamaguchi K., Ota M. (2016). Microcutting using a micro turn-milling machine. Precis. Eng..

[5] Trent E.M., Wright P.K. (2000). Metal Cutting.

[6] Lee Y.J., Wang H. (2020). Current understanding of surfaces effect in microcutting. Mater. Des..

[7] Cheng K., Huo D. (2013). Micro-Cutting: Fundamentals and Applications.

[8] To S.S., Wang H., Lee W.B. (2018). Factors influencing machined surface quality. Materials Characterisation and Mechanism of Micro-Cutting in Ultra-Precision Diamond Turning.

[9] Rahman M.A., Rahman M., Kumar A.S. (2018). Influence of relative tool sharpness (RTS) on different ultra-precision machining regimes of Mg alloy. Int. J. Adv. Manuf. Technol..

[10] Rahman M.A., Woon K.S., Venkatesh V.C., Rahman M. (2018). Modelling of the combined microstructural and cutting edge effects in ultraprecision machining. CIRP Ann..

[11] Gomes M.C., Brito L.C., da Silva M.B. (2021). Tool wear monitoring in micromilling using Support Vector Machinewith vibration and sound sensors. Precis. Eng..

[12] Kim J.D., Moon C.H. (1996). A study on microcutting for the configuration of tools using molecular dynamics. J. Mater. Processing Technol..

[13] Wang X.J., Yang Z.R., Xu F.F., Wang L.P. (2019). Improving surface quality in microcutting of B-10/Al composite. Ind. Lubr. Tribol..

[14] Rahman M.A., Amrun M.R., Rahman M., Kumar A.S. (2017). Variation of surface generation mechanisms in ultra-precision machining due to relative tool sharpness (RTS) and material properties. Int. J. Mach. Tools Manuf..

[15] Wu X., Li L., He N., Yao C., Zhao M. (2016). Influence of the cutting edge radius and the material grain size on the cutting force in micro cutting. Precis. Eng..

[16] Yang K., Liang Y., Zheng K., Bai Q., Chen W. (2011). Tool edge radius effect on cutting temperature in micro-end-milling process. Int. J. Adv. Manuf. Technol..

[17] Florea D.A., Pena A.E., Zapciu M. (2017). Surface quality and machining time optimization based on feedrate correction function of tool trajectories types. Tech. Vjesn..

[18] Heidari M., Hosseini S.V., Parvaz H. (2021). Modelling and Optimization of Surface Roughness and Specific Tool Wear in MillingProcess. Tech. Gaz..

[19] Dumitras C.G. (2006). Modeling of deburring process, Modern Practice in Stress and Vibration Analysis VI. Proc. Appl. Mech. Mater..

[20] Cochran W.G., Cox G.M. (1968). Book Experimental Design.

